# Limitations of the MELD score in predicting mortality or need for removal from waiting list in patients awaiting liver transplantation

**DOI:** 10.1186/1471-230X-9-72

**Published:** 2009-09-25

**Authors:** Daniel Gotthardt, Karl Heinz Weiss, Melanie Baumgärtner, Alexandra Zahn, Wolfgang Stremmel, Jan Schmidt, Thomas Bruckner, Peter Sauer

**Affiliations:** 1 Department of Internal Medicine IV, University Hospital of Heidelberg, Im Neuenheimer Feld 410, 69120 Heidelberg, Germany; 2 Department of Surgery, University Hospital of Heidelberg, Im Neuenheimer Feld 110, 69120 Heidelberg, Germany; 3 Institute for Medical Biometry and Informatics, University Hospital Heidelberg, Im Neuenheimer Feld 305, 69210 Heidelberg, Germany

## Abstract

**Background:**

Decompensated cirrhosis is associated with a poor prognosis and liver transplantation provides the only curative treatment option with excellent long-term results. The relative shortage of organ donors renders the allocation algorithms of organs essential. The optimal strategy based on scoring systems and/or waiting time is still under debate.

**Methods:**

Data sets of 268 consecutive patients listed for single-organ liver transplantation for nonfulminant liver disease between 2003 and 2005 were included into the study. The Model for End-Stage Liver Disease (MELD) and Child-Turcotte-Pugh (CTP) scores of all patients at the time of listing were used for calculation. The predictive ability not only for mortality on the waiting list but also for the need for withdrawal from the waiting list was calculated for both scores. The Mann-Whitney-U Test was used for the univariate analysis and the AUC-Model for discrimination of the scores.

**Results:**

In the univariate analysis comparing patients who are still on the waiting list and patients who died or were removed from the waiting list due to poor conditions, the serum albumin, bilirubin INR, and CTP and MELD scores as well as the presence of ascites and encephalopathy were significantly different between the groups (p < 0.05), whereas serum creatinine and urea showed no difference.

Comparing the predictive abilities of CTP and MELD scores, the best discrimination between patients still alive on the waiting list and patients who died on or were removed from the waiting list was achieved at a CTP score of ≥9 and a MELD score of ≥14.4. The sensitivity and specificity to identify mortality or severe deterioration for CTP was 69.0% and 70.5%, respectively; for MELD, it was 62.1% and 72.7%, respectively. This result was supported by the AUC analysis showing a strong trend for superiority of CTP over MELD scores (AUROC 0.73 and 0.68, resp.; p = 0.091).

**Conclusion:**

The long term prediction of mortality or removal from waiting list in patients awaiting liver transplantation might be better assessed by the CTP score than the MELD score. This might have implications for the development of new improved scoring systems.

## Background

Liver transplantation (LTx) provides the only curative treatment option with excellent long-term results in patients with decompensated cirrhosis of the liver [1]. The Child-Turcotte-Pugh (CTP) score [2], originally developed for the assessment of the outcome of patients with cirrhosis and portal hypertension, was extended for general prognosis, and to stratify patients on the waiting list for LTx [1].

The use of CTP in prioritizing potential liver transplant recipients is limited by several factors: the variables, ascites and encephalopathy, are all subjective and are influenced by medical therapy. The lack of an assessment of renal function, which is a reliable prognostic marker in cirrhosis, is an additional limiting factor [3].

The Model for End-Stage Liver Disease (MELD) is a scoring system for the severity of liver disease initially developed as a model in predicting poor survival in patients after transjugular intrahepatic porto-systemic shunt (TIPS). [4,5]. A modification of this score was developed to predict mortality in patients with cirrhosis of different etiologies and severities of liver disease [6]. This MELD score was found to be superior to the CTP score in predicting 3-months mortality and therefore the MELD score was implemented in 2002 in the United States for the prioritization of LTx recipients.

However, the MELD score has been criticized for several different reasons. Some studies have revealed marked variations in serum creatinine measurement when different laboratory methodologies are used [7]. INR was designed to standardize the anticoagulation effect of warfarin and may not reflect the severity of liver disease [8]. Two studies that used different assays to measure INR led to significantly different MELD scores between transplant centers [9].

In countries within the alliance of Eurotransplant, the MELD score for prioritization of patients awaiting Ltx was initiated in November 2006 and at present little information is available concerning the prognostic ability of this allocation system compared to the previous system, which was based on CTP score and waiting time.

In this retrospective analysis, we evaluate the MELD score in comparison to the CTP score in order to better assess the prognostic ability of these different methods in predicting mortality on the waiting list as well as the need for removal from waiting list due to deterioration of the overall clinical condition.

## Methods

Data sets of 268 consecutive patients listed for single-organ LTx for nonfulminant liver disease between 2003 and 2005 were included in the study. Baseline characteristics of patients included age, gender, body mass index, etiology of liver disease and clinically relevant comorbidity (Table 1).

**Table 1:** Baseline characteristics parameters at the time of listing

	N/Mean	SD, (Range),%	95% CI of mean
Patients	268		

Age (years)	50.54	11.32 (16 - 68)	49.2-51.9

Female/Male	99/169		

BMI	25.1	4.5 (14.5 - 40.4)	24.5-25.7

Etiology of liver disease			
Cirrhosis - alcoholic	79	29.5	
Cirrhosis - viral	75	28.0	
other	54	20.2	
malignancy	39	14.5	
Cholestatic liver disease	21	7.8	

Co-morbidity			
Diabetes	62	23.1	
Coronary heart disease	10	3.7	
Hypertension	41	15.3	
Renal insuffiency	34	12.7	
HRS	15	5.6	
Ascites	70	26.1	
HE	78	29.1	

MELD score	14.2	6.4 (6.4 - 40)	13.4-14.9

CTP score	8.0	8.0 (5 - 14)	7.8-8.3

(N = 20) patients who underwent re-transplantation for recurrent disease with cirrhosis after previous transplantation were included in the trial, whereas patients with early graft failure without cirrhosis of the graft at the time of listing for re-transplantation were not included.

The CTP includes two clinical variables, ascites and encephalopathy, and three laboratory parameters, serum bilirubin, albumin and prothrombine time. Each variable is scored from 1 to 3 with the sum of each scored variable representing the CTP score. The MELD score was calculated using the model previously described [6]. MELD and CTP scores for all patients at the time of listing were used for calculation.

The predictive abilities of CTP and MELD scores not only for mortality on the waiting list but also for the need for withdrawal from the waiting list were calculated.

All analyses were performed using SPSS 16.0 (SPSS Inc. Chicago, IL). The Mann-Whitney-U Test was used for the univariate analysis and the AUC-Model for discrimination of the scores.

The study protocol conformed to the ethical guidelines of the Helsinki Declaration, and was approved by the ethics committee of the University of Heidelberg.

## Results

Clinical characteristics and demographic data of patients included in the study are shown in Table 1. The mean age of patients was 50.5 years (range 16 to 68 years). Of the 268 patients, 99 were female and 169 were male. The mean body mass index was 25.1 (range 14.5-40.4). Approximately one-third of patients suffered from alcoholic liver disease and another one-third from virus hepatitis-induced cirrhosis, respectively. Malignancies and cholestatic liver disease accounted for 14.5% and 7.8%, respectively. Other etiologies accounted for 20.2%.

Ascites, encephalopathy and hepatorenal syndrome as complications of liver cirrhosis were noted in 26%, 29% and 5.6% of patients, respectively. Other comorbidity factors not associated with the underlying liver disease were diabetes (23.1%), coronary heart disease (3.7%), hypertension (15.3%) and renal failure other than hepatorenal syndrome (12.7%).

The mean time on waiting list for the whole cohort of patients was 357 days (range 9 to 1836 days). 37 patients were removed from the waiting list: 23 patients died, six patients were removed due to poor conditions, six patients improved and liver transplantation was no longer considered to be indicated, and two patients retracted their agreement to transplantation. The 23 patients who died and the 6 patients who were removed for poor conditions were included into the calculation for the predictive ability of the two scores (Table 2).

**Table 2:** Events on waiting list

	n	% t
Days on waiting list	357 ± 328 (9-1836)	328 1

Removed from waiting list	37	22.0
Died	23	13.7
Poor	6	3.6
Better	6	3.6
Other	2	1.2
Mortality and removed due to poor conditions		17.2

Tx	100	37.3

During the study period, 100 of the 268 patients were successfully transplanted.

The mean MELD score of all patients was 14.2 (range 6.4 to 40), the mean CTP score was 8.0 (range 5 to 14).

In the univariate analysis comparing patients still on the waiting list and patients who died or were removed due to poor conditions, serum albumin, bilirubin INR, and CTP and MELD scores as well as the presence of ascites and encephalopathy were significantly different between the groups (p < 0.05), whereas serum creatinine and urea were not different (Table 3).

**Table 3:** Mortality on waiting list or removed from waiting list due to poor conditions (patients with Tx excluded) univarate analysis

	Still on waiting list (n = 139)	Died or removed from waiting list (n = 29)	P value
	**Mean ± SD (95% CI)**	**Mean ± SD (95% CI)**	

Albumin [g/l]	34.9 ± 11.3 (33.1-36.8)	24.3 ± 12.4 (19.6-29.1)	0.0001

Bilirubin [mg/dl]	2.9 ± 3.5 (2.4-3.6)	6.5 ± 9.1 (3.0-10.0)	0.004

INR	1.25 ± 0.2 (1.2-1.3)	1.56 ± 0.34 (1.3-1.6)	0.0001

Creatinine [mg/dl]	1.04 ± 0.8 (0.8-1.3)	1.57 ± 3.1 (0.4-2.7)	Ns

Urea [mg/dl]	32.0 ± 18.4 (29.0-35.2)	41.5 ± 26.0 (28.2-54.8)	Ns

CTP score	7.5 ± 1.8 (7.2-7.8)	9.4 ± 2.4 (8.5-10.3)	0.0001

MELD score	13.2 ± 5.9 (12.1-14.1)	17.2 ± 7.4 (14.4-20.1)	0.002

Ascites	1:112 (81%)	1:20 (69%)	Ns
	2:20 (14%)	2:6 (21%)	
	3:7 (5%)	3:3 (10%)	

HE	1:107 (77%)	1:19 (65%)	Ns
	2:31 (22%)	2:9 (31%)	
	3:1 (1%)	3:1 (4%)	

Combined calculation of ascites plus HE showed no difference

Ascites and HE were classified from stage 1 to 3

**Figure 1:**
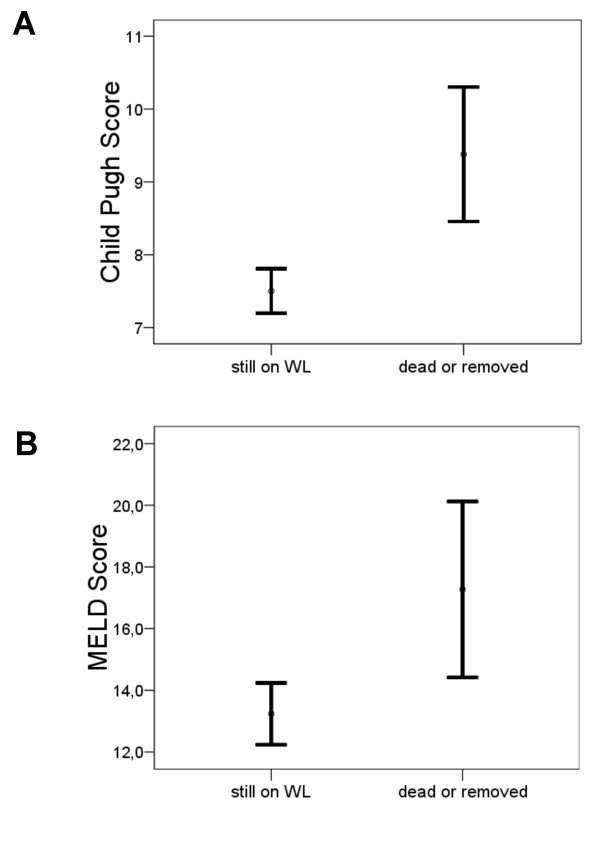
**The errors bars are shown for the discrimination of patients by CTP (A) and MELD (B) scores revealing a better cutoff for CTP**.

Comparing the predictive capability of CTP and MELD scores, discrimination between patients still alive on the waiting list and patients who died or were removed was determined by minimizing the false positive and false negative results of each score. Patient who underwent transplantation during the observation period were excluded for this analysis. Of 29 patients who died or were removed from the waiting list a CTP score of ≥9 identified 20 patients and a MELD score of ≥14.4 identified 18 patients (Figure 1). Of the 139 patients still on the waiting list a CTP score of < 9 correctly identified 98 patients, the MELD score of < 14.4 101 patients. The sensitivity and specificity to identify mortality or severe deterioration was 69.0% and 70.5% for CTP, respectively; MELD achieved only a 62.1% sensitivity and a 72.7% specificity (Tables 4, 5 and 6). Discrimination was achieved by CTP with a p value of 0.00009 versus MELD with a p value of 0.002.

**Table 4:** Differentiation of CTP and MELD scores (no Tx)

	CTP Score <9	CTP Score ≥9	MELD Score <14.4	MELD score ≥14.4
Still on waiting list	98	41	101	38

Died or removed	9	20	11	18

MELD:

Sensitivity 18/29: 62.1%

Specificity 101/139: 72.7%

CTP:

Sensitivity 20/29: 69.0%

Specificity 98/139: 70.5%

**Table 5:** Comparison of CTP and MELD scores: still on waiting list and no Tx

MELD	<14.4	≥14.4	Total
CTP			

<9	84	14	98

≥9	17	24	41

	101	38	

**Table 6:** Comparison of CTP and MELD scores: died on or removed from the waiting list

MELD	<14.4	≥14.4	Total
CTP			

<9	9	0	9

≥9	2	18	20

	11	18	

**Figure 2:**
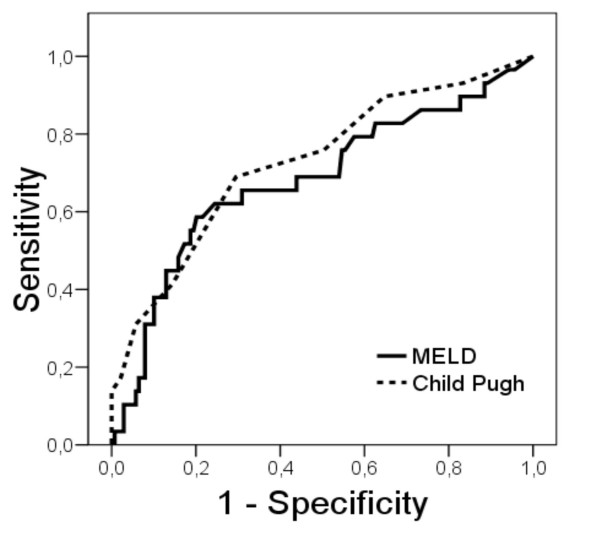
**The ROC curve shows the performance of the CTP score (AUROC 0.73) compared to the MELD score (AUROC 0.68)**.

The area under ROC of the MELD score was 0.68 and of CTP score it was 0.73 (c-statistics; p = 0.091). Although the difference was not statistically significant, a trend to superiority of CTP was observed, supporting the results of the univariate discrimination analysis. (Figure 2).

## Discussion

Increased mortality of patients on the waiting list for LTx and shortage of donor organs gave rise to efforts to improve allocation criteria for liver transplantation candidates. The MELD scoring system has been implemented in United States for prioritization in 2002, and within the alliance of Eurotransplant November 2006.

In this retrospective analysis, we evaluated the MELD score in comparison to the CTP score in order to assess the prognostic ability of these different methods in predicting mortality on the waiting list as well as the need for the removal from waiting list due to deterioration of the overall clinical condition. The latter condition had not been regularly assessed in prior studies evaluating MELD and CTP scores. Interestingly one study which analyzed waiting list mortality in this respect showed similar predictive value of MELD and CTP for waiting list mortality [10].

In our study, which comprised an observation period of approximately one year, MELD was not shown to be superior to CTP. In fact, we found that CTP actually yielded better discrimination of patients for this forecasting horizon. This is in contrast to most other studies, which have focused on a prediction of 3-months mortality. Both scores performed well in discriminating, but in our study a CTP cut off of 9 classified more patients correctly in either the survival group or the mortality/removal group than the best MELD cut off of 14.4. This was reflected by the higher level of significance of CTP compared to MELD. In addition most studies did not take into account patients removed from waiting list due to poor condition. All patients removed from the waiting list in our study due to deterioration of medical status were considered as if they had died while on the waiting list. This appears to be more accurate, since commonly these patients do not return to the waiting list condition. However, the fact that the better discrimination was achieved only in the univariate analysis, whereas the c-statistics showed a strong trend, but did not reach level of significance may limit the conclusions of our retrospective evaluation.

Previous studies designated MELD scores as being better prognostically for LTx. A prospective study of more than 3000 adult pre-LT patients with chronic liver disease examined the outcome over a 3-months period. Within three months of listing, 12% had died, and the 3-month mortality was significantly larger in patients with higher MELD scores. The c-statistic for 3-month mortality in this study was 0.83 for the MELD score, significantly better in comparison to 0.76 for the CTP score [11]. Another prospective study evaluated baseline MELD and CTP scores to predict 6-month mortality and 12-month mortality. MELD discriminated better in this study as well [12]. However, several studies have also failed to confirm the superiority of the MELD score compared to the CTP score for patients on the waiting list. In the evaluation of the hitherto largest cohort of almost 7000 patients with end stage liver disease (listed as status 2A, 2B, and also status 3) showed that the CTP was slightly better, but not significantly different than MELD score in predicting 3-month survival. This is somewhat surprising since these authors extracted their data from the same UNOS database as the one used in a previous trial [11]. In this study, status 3 patients were included, perhaps indicating that patients with compensated cirrhosis are not adequately classified by MELD [13]. This would be in line with our study, which included status 3 patients as well. But in our study, which analyzed a period of about one year, the CTP score performed better than MELD in predicting death or removal from waiting list. The data sets used were generated while waiting time was a major factor for organ allocation within the alliance of Eurotransplant and MELD score had not been implemented, this might explain the fact that the mean MELD score of our study group at the time of listing was comparatively low. During waiting time MELD score increased then to levels, at which patients benefit from transplantation [14].

Considering these 11 published studies, only four demonstrated a statistical superiority of MELD over the CTP score (approx. 4,500 patients), whereas seven showed no statistical differences (approx. 8,000 patients). However, no study showed MELD to be statistically inferior to CTP score.

The MELD score has the advantage that it is based on a multivariable analysis of objective tests for serum bilirubin, INR and serum creatinine. Compared to CTP score, it also includes assessment of renal function, another major marker of the severity of the disease. Though serum bilirubin, creatinine, and INR are usually considered objective and therefore highly reliable, they may also be influenced by therapeutic manipulations, not only by disease progression. So one important advantage of MELD, namely the independence of the subjective judgment by a clinician, is counterbalanced in part by arbitrary laboratory values. In our analysis creatinine was not usable in discriminating patient groups. This might be due to the fact, that impaired renal function only plays a major role in short-term horizons. The very short life expectancy of patients with hepatorenal syndrome is well characterized. Or it could be due to the fact that creatinine is not a very good marker for renal function in these patients due to low body mass and partly peritoneal dialysis in decompensated ascites.

Several attempts to improve the predictive ability of MELD score were made by adding clinical variables (hepatic encephalopathy, ascites) or laboratory parameters (sodium), [15-18], the latter being the most promising [19], and the former forfeiting the benefit of objective parameters.

Another effort to improve MELD involved analyzing the change in MELD scores over time, bearing in mind that this dynamic variable would reflect the dynamic of disease in this patient. In a retrospective evaluation of 760 patients the delta MELD score had better prediction ability for mortality than the baseline MELD score [20]. However, a retrospective evaluation found the delta MELD score to be less predictive compared with the most up-to-date MELD score [21]. In another study, the delta MELD score per month at 6 and 12 months was significantly better compared with baseline the MELD and CTP [12]. In our trial, the change in MELD score was not superior in predicting survival or the need for removal from the waiting list compared to baseline MELD (data not shown).

In comparison to other studies, we analyzed not only death on the waiting list but also well removal from the waiting list due to poor condition; additionally, we extended the patient observation time over a period of one year. This may help to explain the differences of our results compared to other studies. But more importantly, from our point of view, this combined end point more accurately reflects the natural history of disease and its reality in our center. However, the MELD score is established for the 3 months period, which may be the most important time frame for allocation. Our data do not argue against the use of MELD concerning priorization of patients during the initial period on the waiting list. But in patients with a longer time on waiting list CTP may serve as an additional factor for assessment of patient prognosis. Furthermore, since our data suggest that some aspects of prognosis of cirrhosis are better reflected by CTP score, they might assist in the development of new scoring systems for allocation.

## Conclusion

The increasing numbers of standard exceptions for MELD scores, for example cholestatic diseases, reflect the clinical need to improve this allocation system. Although our study does not argue against the use of the MELD score for short term allocation of organs and priorization of recipients, the long term prediction of mortality or removal from waiting list in patients awaiting liver transplantation might be better assessed by the CTP score than the MELD score. This might have implications for the development of new improved scoring systems.

## List of abbreviations

CTP: Child-Turcotte-Pugh; LTx: liver transplantation; MELD: Model of End-Stage Liver Disease; TIPS: transjugular intra-hepatic porto-systemic shunt.

## Competing interests

The authors declare that they have no competing interests.

## Authors' contributions

DG took part in designing the study, analyzing the data and writing the manuscript. KHW took part in designing the study, gathering the data and writing the manuscript. MB took part in gathering the data and writing the manuscript. AZ took part in gathering the data and writing the manuscript. WS and JS took part in designing the study and writing the manuscript. TB took part in statistical analysis and writing the manuscript. PS took part in designing the study, analyzing the data and writing the manuscript. All authors read and approved the final manuscript.

## Pre-publication history

The pre-publication history for this paper can be accessed here:

http://www.biomedcentral.com/1471-230X/9/72/prepub
